# Acclimation of functional traits leads to biomass increases in leafy green species grown in aquaponics

**DOI:** 10.1093/aobpla/plaf005

**Published:** 2025-01-20

**Authors:** Victoria Nicholes, Malik Khan, Nicholas Lemon, Peter Vila, Courtney Campany

**Affiliations:** Department of Natural and Physical Sciences, Shepherd University, 301 N. King St., Shepherdstown, WV, 25443, USA; Department of Biology, West Virginia University, Life Sciences Bldg, PO Box 6057, Morgantown, WV, 26506, USA; Department of Natural and Physical Sciences, Shepherd University, 301 N. King St., Shepherdstown, WV, 25443, USA; Department of Natural and Physical Sciences, Shepherd University, 301 N. King St., Shepherdstown, WV, 25443, USA; Department of Natural and Physical Sciences, Shepherd University, 301 N. King St., Shepherdstown, WV, 25443, USA; Department of Natural and Physical Sciences, Shepherd University, 301 N. King St., Shepherdstown, WV, 25443, USA

**Keywords:** aquaponics, photosynthesis, plants, nitrogen, stomata, water-use efficiency

## Abstract

As human population size continues to increase and climate change effects worsen, future food security has become a primary concern for agricultural industries worldwide. Yields of traditional agricultural methods are commonly limited by water and nutrient availability and many crop yields are predicted to decline. Alternative farming practices like aquaponics, which can alleviate these negative yield pressures, may become critical to reaching food production targets. Aquaponics approaches involve the cyclic joint production of fish and hydroponic plants where the fish efflux provides nutrients to plants that then purify the water to be recycled to the fish tanks. In this study, we investigated the acclimation of physiology and functional traits of plants grown in aquaponics versus soil for three leafy green species. We compared gas exchange, stomatal anatomy, water-use efficiency, and foliar chemistry on newly formed leaves across weekly measurements. Increased photosynthetic rate, driven by higher stomatal conductance and increases in tissue nitrogen, led to higher biomass production in aquaponics for all species. Aquaponics plants adjusted stomatal behavior and to a lesser degree stomatal anatomy to become less water-use efficient than plants grown in soil. Collectively, our findings demonstrate the ability of plants to acclimate quickly to aquaponics growing systems that largely remove water and nutrient limitations to plant growth. The increased biomass production of broccoli, pak choi, and salanova by 185%, 116%, and 362% in aquaponics compared to soil-grown plants demonstrates the potential of small-scale aquaponics systems as an efficient and sustainable alternative farming practice.

## Teaser

As aspects of our food system are under pressure from population growth and global change, the need to investigate the efficacy of alternate agriculture practices is imminently important. Our study examined the capacity of leafy green species grown in aquaponics to acclimate to unlimited access to water and consistent supplies of nitrogen from fish efflux, compared to the same species grown in soil. We found biomass production, rates of photosynthesis, and stomatal conductance to be higher in aquaponics, while water use efficiency was lower in aquaponics for all species. Our study also found nitrogen supply to be higher in leaf tissues in plants grown in aquaponics, which correlated with the observed higher rates of photosynthesis. Our findings offer mechanistic insights into the future of sustainable farming that utilizes aquaponics to produce non-staple crops that meaningfully contribute to feed insecurity.

## Introduction

### Global food security

The human population is predicted to surge to over 9 billion by 2050 (Roberts 2011; Cleland 2013; Cole *et al.* 2018; Mote Rivas and Kalnay 2020; Siegel 2021) making sufficient food production to meet global needs an urgent problem. Within the next few decades, production of the major grain-producing staple crops including maize, wheat, and rice will need to increase by 50%–70% to feed the expanding population (Cairns *et al.* 2013; Ray *et al.* 2013; Tai Martin and Heald 2014; Long Marshall-Colon and Zhu 2015; Rockström *et al.* 2017; Chouchane Krol and Hoekstra 2018; Asseng *et al.* 2020; Gerten *et al.* 2020). Food security has not improved by increases in cropland efficiency as crop nutritional value has not followed the increase of production and converting natural lands to croplands has led to detrimental losses in biodiversity, soil health, and ecosystem stability (Clark and Tilman 2017; Ramankutty *et al.* 2018).

Additionally, biomass yields of some non-staple and legume crops are decreasing due to limited water supply and climate warming (Fahad Ali A Bajwa *et al.* 2017; Zhou *et al.* 2017; Bisbis Gruda and Blanke 2018); but impacts of climate change factors on production of non-staple crop impacts are less studied (Zhou *et al.* 2017; Scheelbeek *et al.* 2018). Further, global change factors such as pest introductions, shifts in pest phenology, and pest range expansion place additional negative pressures on crop productivity and food security (Boyer *et al.* 2013; Bebber Holmes and Gurr 2014; Nabout *et al.* 2016). These impacts will further decrease harvests and increase the urgency to enhance crop productivity to ensure food security (Boyer *et al.* 2013; Nabout *et al.* 2016; Hanes and Rhode 2050).

Globally, staple crops are impacted by nutrient and water deficiencies that restrict crop growth (Mueller *et al.* 2012). For example, nutrients and water can limit production of staple crops, such as wheat, soybean, and maize production by greater than 45% (Fahad Ali A. Bajwa *et al.* 2017). Biomass production limitations are often linked to anthropogenic disturbances like soil erosion outpacing the rate of soil replenishment in plowed fields that results in the loss of key soil nutrients such as nitrogen and phosphorus (Yuan *et al.* 2018; Dror Yaron and Berkowitz 2021) along with micronutrients like selenium (Jones *et al.* 2013). Additionally, the use of heavy agricultural machinery paired with other human field traffic destroys soil aggregates and degrades soil pore structure that can stunt root growth and decrease biomass production by as much as 50% (Shah *et al.* 2017). Additionally, alleviation of nutrient and water limitations in lettuce, an important non-staple crop, can improve production by more than 130% (Wang *et al.* 2023).

With altered soil nutrient cycling, the agriculture industry heavily relies on fertilizers to supplement these growth-limiting nutrients (Grzebisz *et al.* 2013; Paulot and Jacob 2014; Houlton *et al.* 2019) but it is difficult to balance the environmental harm of unstainable fertilizer use on enhanced agricultural production (X. Zhang *et al.* 2015). Fifty percent of the added nitrogen fertilizer in conventional soil agriculture ends up in the harvested biomass with the remaining 50% susceptible to nitrate leaching into groundwater or runoff, or as ammonia or nitrous oxide emissions to the atmosphere (Philip Robertson *et al.* 2013). These permanent removals of elemental resources via harvesting biomass and erosion drive a continuous, accelerating feedback loop towards poor soil quality, increased fertilizer use, and further environmental damage (Davidson *et al.* 2015). Moreover, other soil constraints such as low moisture content and high salinity contribute to biomass production shortfalls by constraining plant responses to vapor pressure deficits, disrupting ion homeostasis, reducing photosynthesis, and diverting energy for growth towards soil water uptake (Cemek *et al.* 2011; Zörb Geilfus and Dietz 2019; Rigden *et al.* 2020).

Groundwater depletion makes any increase in irrigated croplands unsustainable (Dalin *et al.* 2017; Perrone and Jasechko 2017) and projected increases in drought across major agriculture zones coupled with scarce groundwater supplies reduce the potential of irrigating new lands (Peña-Gallardo *et al.* 2018; Leng and Hall 2019). The complexity of tracking consumption, restricting extractions, and negotiating with irrigators makes increasing current irrigation efficiency an ineffective, slow solution (Grafton *et al.* 2018). To overcome water concerns, farming industries are turning to altering irrigation practices like plant-centric irrigation (Zhang *et al.* 2021) and water-saving irrigation (Xu *et al.* 2015b). However, reliance on irrigation to solve the climate water crisis is impossible with current renewable water resources falling short of the irrigation demand by as much as 47% (Wang *et al.* 2021).

### Photosynthetic constraints of crops

Photosynthesis is fundamental to the production of food as it generates the raw materials for all plant products (Richards 2000). Photosynthesis in plants is tied to species-specific leaf traits such as leaf nitrogen content that regulates photosynthetic capacity and sensitivity to water stress through transpiration rate and stomatal conductance that regulates stomatal behavior. Photosynthesis requires many proteins, especially Rubisco, that account for the majority of nitrogen content in leaves (Evans and Clarke 2019). Photosynthesis is well-known to be limited by nitrogen availability, especially because photosynthetic enzymes require high nitrogen investments to build and maintain (Evans 1989; Evans and Seemann 1989). In addition, under water restriction, plants must optimize stomatal behavior to minimize transpiration losses while maximizing the CO_2_ drawdown needed to drive photosynthetic reactions. Current and projected climate instability, including higher temperatures paired with decreasing precipitation, drives increased stomatal closure via changes in soil hydraulic conductivity and higher vapor pressure deficits (Carminati and Javaux 2020; Grossiord *et al.* 2020; Abdalla *et al.* 2022). Overall, fluctuating levels of water stress and nitrogen availability drive changes in photosynthetic traits that vary widely between species (Ahanger *et al.* 2016; Wang Wang and Shangguan 2016; Richards 2018).

Bioengineering and genetic manipulation research focused on drought tolerance and nutrient deficiency traits has increased photosynthetic rates and crop yields (Bouzid *et al.* 2019; Conti 2019; Condon 2020; Martignago *et al.* 2020; Dietz Zörb and Geilfus 2021; Ozeki Miyazawa and Sugiura 2022). However, these approaches are often limited by unexpected side effects of inserting transgenes and biological constraints of limited availability of genetic resources (Ladics *et al.* 2015; Kumar *et al.* 2020). Improved traditional agriculture techniques like cover cropping (Rosa-Schleich *et al.* 2019), permaculture (Suh 2014), and biodynamic farming (Santoni *et al.* 2022) practices will likely still fall short of our rising food targets. Shortfalls in these improvements to traditional agriculture have driven the refinement of alternative soilless farming methods like aeroponics, hydroponics, and aquaponics (AlShrouf 2017). Aquaponics, a highly efficient system with low pollution and water consumption, combines hydroponics and aquaculture (controlled production of aquatic organisms) and is one of the highest-yield animal production systems (Palm *et al.* 2018; Wei *et al.* 2019). In aquaponics systems, fish waste efflux supplies a hydroponic source of nitrogen-based nutrients to plants, which eliminates water stress and potentially decreases nitrogen limitation for leaf photosynthetic functional traits. Aquaponics plants subsequently filter the fish efflux, which is then recycled to the fish tanks, vastly reducing water requirements (Janni and Jadhav 2022). Although soilless growth systems are shown to increase the yield and quality of some crops compared to soil (Verdoliva *et al.* 2021), the limited spatial capacity of those systems to contribute to crop yield demands is largely untested. Consequently, soilless agricultural practices, like aquaponics systems with cyclic water and nutrient renewal from fish efflux, need to become a research priority.

The objective of this study is to compare leaf-level functional traits in plants grown in soil to plants grown in aquaponics to investigate the capacity of aquaponics plants to acclimate to the resource environment created by fish efflux and hydroponics. We used three leafy green crop species, common in aquaponics systems, to test for acclimation of leaf gas exchange, stomatal anatomy, water-use efficiency, and foliar chemistry on newly formed leaves. We hypothesized that plants in an aquaponics system will acclimate stomatal behavior and anatomy to upregulate photosynthesis despite the increased water cost of carbon gain via transpiration.

## Methods

### Study site

The Shepherd University Aquaponics Laboratory is located at Shepherd Farm, a 160-acre agricultural innovation center for teaching and demonstrating small-scale agricultural techniques, in Shepherdstown WV, USA (39.44549°N, 77.83048°W). The indoor aquaponics facility uses two 4542-liter fish tanks that provide fish efflux to separate hydroponic plant growth systems (Supplementary Figure S1). This study utilized the nutrient water from the Tilapia tank that contains 60 *Oreochromis aureus* fed with Purina Aquamax Pondfish 4000 (Nestlé Purina PetCare, St. Louis, Missouri, USA). The large particulate fish waste from the tank is removed in the settling tank with the excreted ammonia converted to nitrate in the biofilter. Fish tank temperature ranged from 24°C to 25°C with a pH of 7.5–8.0. Water from the Deep-Water Culture beds (DWC) flows to a sump tank and is pumped back into the fish tanks. The water flows at a rate of ~3785 liters per hour through the growing systems. Aquaponic plants were grown in the DWC beds that were 1.2 m wide × 5.0 m long × 0.15 m deep. Each bed fits eight floating Styrofoam raft boards (0.6 m × 1.2 m each) that contain 18 2.5 cm. square cutouts. Nitrate levels were measured twice over the study, varied negligibly, and averaged 11.2 mg/l.

### Study design

We tested three commonly used species in aquaponics, *Brassica oleracea var. italica* (broccoli), *Brassica rapa subsp. chinensis* (pak choi), and *Lactuca sativa* (salanova). Plants in the aquaponics treatment were seeded in a 60:40 Coco Coir:Vermiculite media into 2.54 cm. net pots. Each net pot fit into the square cutouts in each floating raft, allowing roots to be exposed to fish efflux from the tilapia tank. Plants in the soil treatment were sowed directly into square 10 cm diameter × 8 cm deep pots with organic topsoil. Pots were placed on top of adjacent floating rafts at the same planting density and spacing (max 18 plants per raft). This ensured that the two experimental groups experienced similar airflow, microclimates, and light conditions. Three 650-watt Scynce Raging Kush II LED lights (ScynceLED, Mesa, Arizona, USA) were mounted directly above the floating rafts, with a 12:12 hour daylight and night cycle light regime with the Cool and Red channels set at 100%. Incident light Photosynthetic Photon Flux density, measured with a Licor 250-A with a LI-193 sensor (LI-COR Biosciences Inc. Lincoln, Nebraska, USA), averaged 410 μmol/m^2^/s at 15 cm above the floating raft. This irradiance level is within ranges shown to increase biomass accumulation, while remaining near the light saturation point of photosynthesis for leafy green species (Wong *et al.* 2020). For each species, we conducted two experimental trials that ran for 3–4 weeks with 10 individuals for each treatment. Trials with broccoli were run simultaneously, while pak choi and salanova trails were run consecutively during the spring and fall semesters, respectively. Broccoli trials occurred from April 19 to May 3, 2022, pak choi from February 8to March 29, 2022, and salanova from October 19 to November 23, 2021. Measurements were initiated when plants had fully formed leaves, approximately 3 weeks for each species. Growth trials were kept relatively short to prevent plants grown in soil from becoming root bound or nutrient limited in small pots. Plants in soil were watered every Monday, Wednesday, and Friday to field capacity and measurements were taken one day after watering. At the end of the trials, all plants were harvested to measure biomass growth. For the soil treatment, plant roots were carefully removed from the soil and washed to remove all soil particles. The dry biomass of shoot and root components of each plant were weighed after oven drying at 60°C to a constant mass.

### Leaf gas exchange

Weekly sampling was conducted on two newly formed leaves; one leaf for gas exchange and the second leaf for stomatal anatomy and stoichiometry. The species used in this experiment produced new, fully formed leaves between weekly measurements. Gas exchange was measured with a LI-6800 Portable Photosynthesis System (LI-COR Biosciences Inc. Lincoln, Nebraska, USA), fitted with a 1 × 3 cm cuvette. Gas exchange measurements were conducted with chamber conditions of *T*_air_ at 20°C, 420 µmol mol^−1^ reference [CO_2_], 60% humidity, photosynthetically active radiation of 1500 mmol m^−2^ s^−1^, and internal lights set to a 9:1 red to blue light ratio. Once water vapor and [CO_2_] values stabilized within the sample chamber, gas exchange parameters including light-saturated rates of photosynthesis (*A*_n_), stomatal conductance (*g*_s_), and transpiration (*E*) were logged once for each leaf. Intrinsic water use efficiency (WUE_g_) was calculated as *A*_n_ divided by *g*_*s*_.

### Foliar stoichiometry and anatomy

For elemental and isotope analysis, representative subsamples of dried leaf and root samples from the final harvest were ground to a fine powder using a Bead Ruptor 96 (OMNI International, Kennesaw, Georgia, USA). Elemental tissue contents (carbon and nitrogen) and shoot δ^13^C were measured with a Carlo Erba NA elemental analyzer coupled with a Thermo Delta C IRMS (Thermo Fischer Scientific, Waltham, Massachusetts, USA). Percentages of carbon and nitrogen in samples calculated by comparison with certified standards and tissue nitrogen (*N*) is reported on a mass basis (g g^−1^). Isotopic signatures of dry matter are reported relative to standard Vienna Pee Dee Belemnite.

Stomatal peels were taken weekly for stomata anatomical traits on the same age cohort of leaves used for gas exchange measurements. For each peel, a liquid bandage (New Skin, Advantice Health New Jersey, USA) was applied on the abaxial side of the leaf and allowed to dry. Clear tape was used to remove and transfer each peel onto a microscope slide. Two peels (~8 cm^2^) were taken from random locations on the fully formed leaf not chosen for gas exchange. Stomatal density (SD, # mm^-2^) was measured by counting stomata under 400× magnification (field of view diameter 0.50 µm) for salanova and 1000× magnification (field of view diameter 0.2 µm) for broccoli and pak choi. For each leaf, SD was counted three times for randomly selected non-overlapping fields of view across the two available peels.

Ten stomata were imaged in either one or two peels per leaf for each plant during weekly measurement trials. All stomatal pictures were taken at 400× magnification. Stomatal size was measured using ImageJ (NIH, Bethesda, Maryland, USA). The width of both guard cells (mm) was measured from the edge of the stomatal aperture to the outside edge of each guard cell. Stomatal length (mm) was measured from the top to the bottom edge of each stomata. The combined stomatal width was multiplied by stomatal length to calculate stomatal size (mm^2^) as in (Franks and Beerling 2009).

### Statistical analyses

A Welch’s *t*-test was first used to compare differences in treatments for each weekly measurement campaign. Across the weekly campaigns, data for all variables were consistently different between treatments. Thus, we report statistical comparisons (*t*-tests) of treatments as pooled data for each species. To investigate acclimation, a two-way repeated measures ANOVA (Type II) was performed to evaluate the effect of treatment (aquaponics or soil) and time (weekly measurements on fully developed leaves) on functional traits and gas exchange parameters for each species separately. The “car” (Fox and Weisberg 2018) package was used to extract model coefficients. Post-hoc pairwise comparisons were computed with the “emmeans” package (Lenth *et al.* 2022) to determine when significant differences developed within a treatment and across treatments. For pairwise comparisons across treatments, we primarily focused on identifying when treatment comparisons diverged (e.g. week 2) for a given variable to evaluate trait acclimation.

To examine bivariate trait relationships between treatments, responses of dependent variables were analyzed with either linear mixed-effect models or generalized additive models (GAM), with treatment as categorical fixed effects and species as a random effect. Akaike Information Criterion scores were used to evaluate and select the best fit model. For linear relationships, differences in slopes of significant relationships between bivariate traits by treatment or species were tested by calculating estimated marginal means and computing pairwise comparisons with the “emmeans” package (Lenth *et al.* 2022). Explained variance (*R*^2^) of mixed models were computed as in Nakagawa and Schielzeth (2013), in which the marginal *R*^2^ represents variance explained by fixed factors and the conditional *R*^2^ represents variance explained by both fixed and random factors. For non-linear trait relationships, confidence intervals were estimated by fitting a generalized additive model to the data with the “mgcv” package (Wood 2017). For significant GAM fits, we report the percent deviance explained and significance of smooth terms. All tests of statistical significance were conducted at an α level of 0.05. All analyses were performed with R 4.2.2 (R Development Core Team 2017).

## Results

### Plant growth

**Figure 1.**
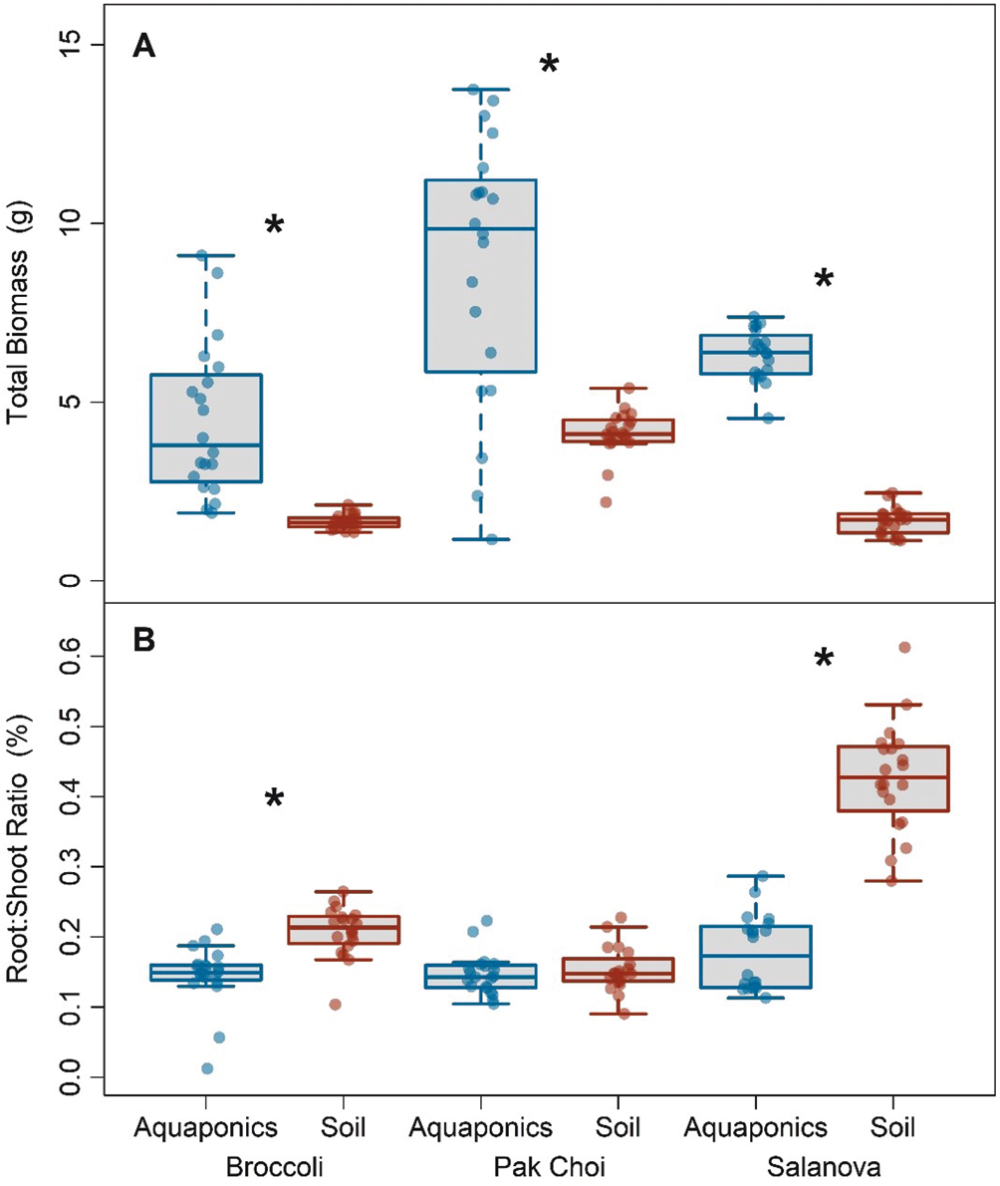
Boxplots of total biomass (A) and root:shoot ratios (B) for three leafy green species in aquaponics and soil treatments. Asterisks represent significant differences between growth treatments for a given species. Each box represents the interquartile range, the line is the median, and the whiskers extend to the lower and upper limits of the data.

Harvested total biomass was significantly greater in aquaponics compared to soil for each species. Total biomass for broccoli, pak choi, and salanova increased by 170%, 114%, and 279%, respectively, in aquaponics (Fig. 1A, all *P* values 0.001). In broccoli, pak choi, and salanova, aboveground shoot biomass increased by 185%, 116%, and 362%, respectively when grown in aquaponics compared to soil (Supplementary Table S1 all *P* values < 0.001). Similarly for roots, broccoli, pak choi, and salanova biomass increased in aquaponics by 96%, 102%, and 86% (Supplementary Table S1, all *P* values < 0.001). The ratio of roots to shoots in broccoli and salanova decreased by 33% and 58%, respectively, in aquaponics treatments (both *P* < 0.001), while biomass partitioning did not change in pak choi (Fig. 1B).

### Photosynthetic parameters

**Figure 2.**
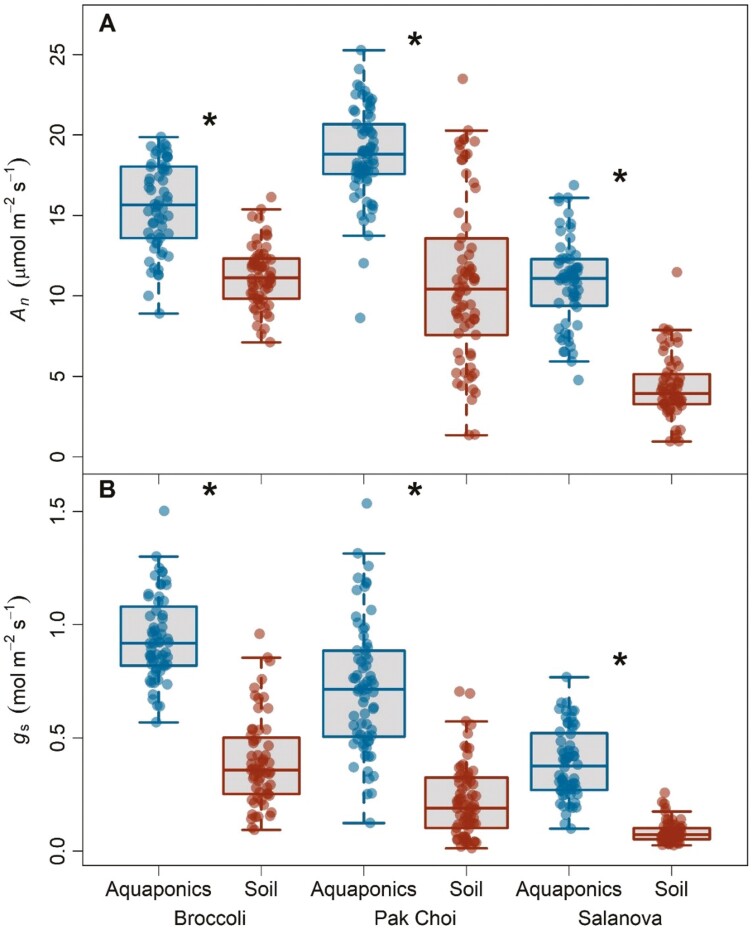
Box plots of (A) light saturated photosynthesis rates (*A*_*n*_) and (B) stomatal conductance (*g*_*s*_) for three leafy green species in aquaponics and soil treatments. Asterisks represent significant differences between growth treatments for a given species. Each box represents the interquartile range, the line is the median, and the whiskers extend to the lower and upper limits of the data.

Photosynthetic parameters broadly changed for all species between aquaponics and soil treatments (Supplementary Table S2). Overall, *A*_*n*_ for broccoli, pak choi, and salanova increased by 39%, 71%, and 153%, respectively, in aquaponics compared to soil treatments (Fig. 2A, *P* < 0.001). For broccoli and salanova, *A*_*n*_ declined in the third week for both treatments (*A*_*n*_ * week, *P* < 0.001). In pak choi, photosynthesis decreased after the first week in soil treatments only (*A*_*n*_ * week, *P* = 0.005).

Overall, *g*_*s*_ increased in aquaponics by 138%, 219%, and 357% in broccoli, pak choi, and salanova, respectively (Fig. 2B, *P* < 0.001). Stomatal conductance in broccoli was variable between weeks in soil treatments only (*g*_s_ * week, *P* < 0.001). For pak choi, stomatal conductance declined after week one for both treatments (*g*_*s*_ * week, *P* = 0.004). In salanova, stomatal conductance declined in week three in aquaponics treatments only (*g*_*s*_ * week, *P* = 0.037).

**Figure 3.**
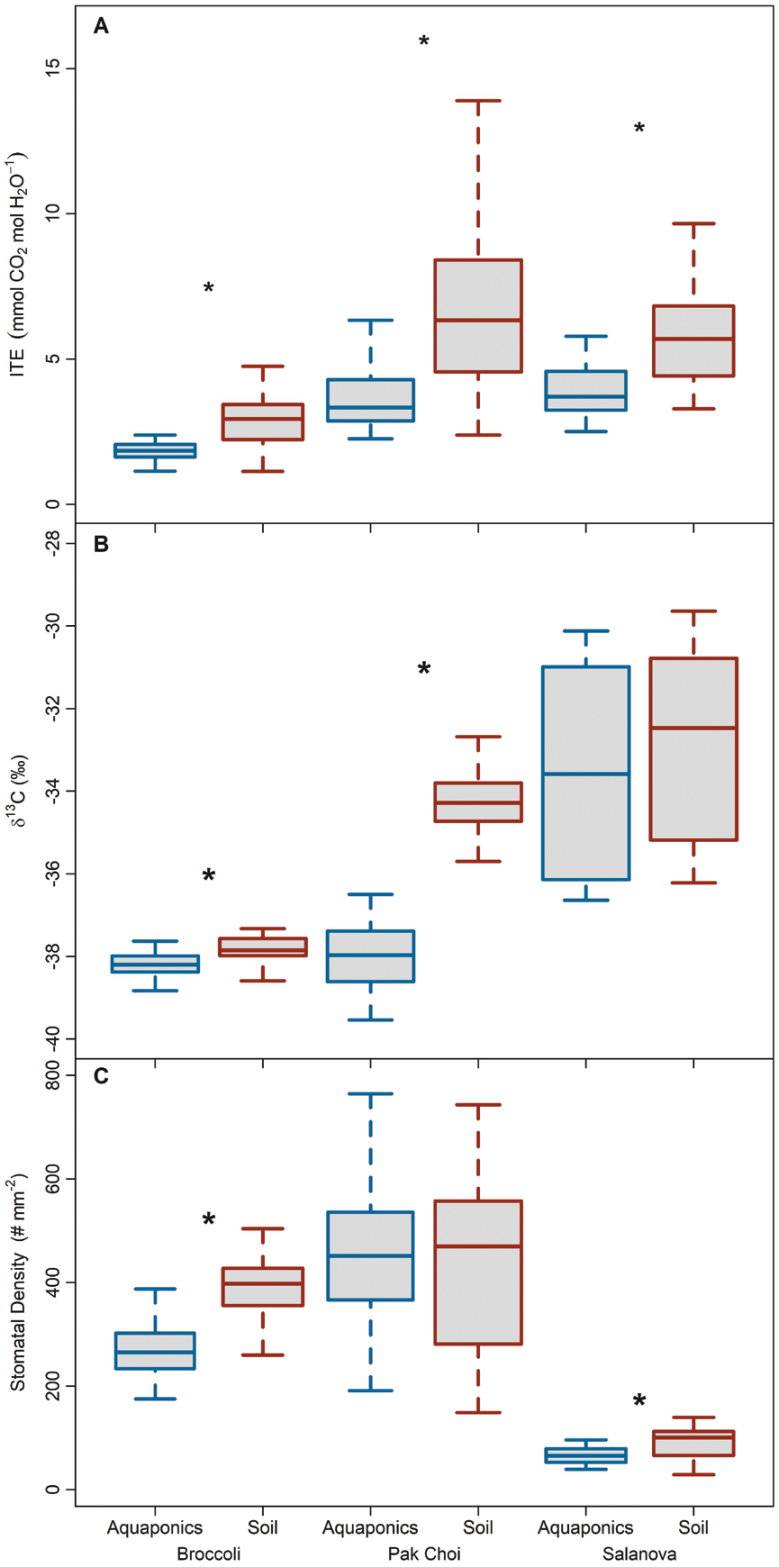
Box plots of (A) intrinsic water use efficiency (WUE_g_) from gas exchange, (B) bulk shoot δ^13^C from harvested aboveground biomass and (C) stomatal density for three leafy green species grown in aquaponics and soil treatments. Asterisks represent significant differences between growth treatments for a given species. Each box represents the interquartile range, the line is the median, and the whiskers extend to the lower and upper limits of the data.

Intrinsic water use efficiency (WUE_g_) decreased significantly in aquaponics by 52%, 54%, and 44% in broccoli, pak choi, and salanova, respectively, compared to soil treatments (Fig. 3A, *P* < 0.001). Intrinsic water-use efficiency in salanova, decreased by week three in aquaponics treatments only (WUE_g_ * week, *P* = 0.037). For broccoli, WUE_g_ was variable from week to week in soil treatments only (WUE_g_ * week, *P* < 0.001).

### Leaf stoichiometry and anatomy

**Figure 4.**
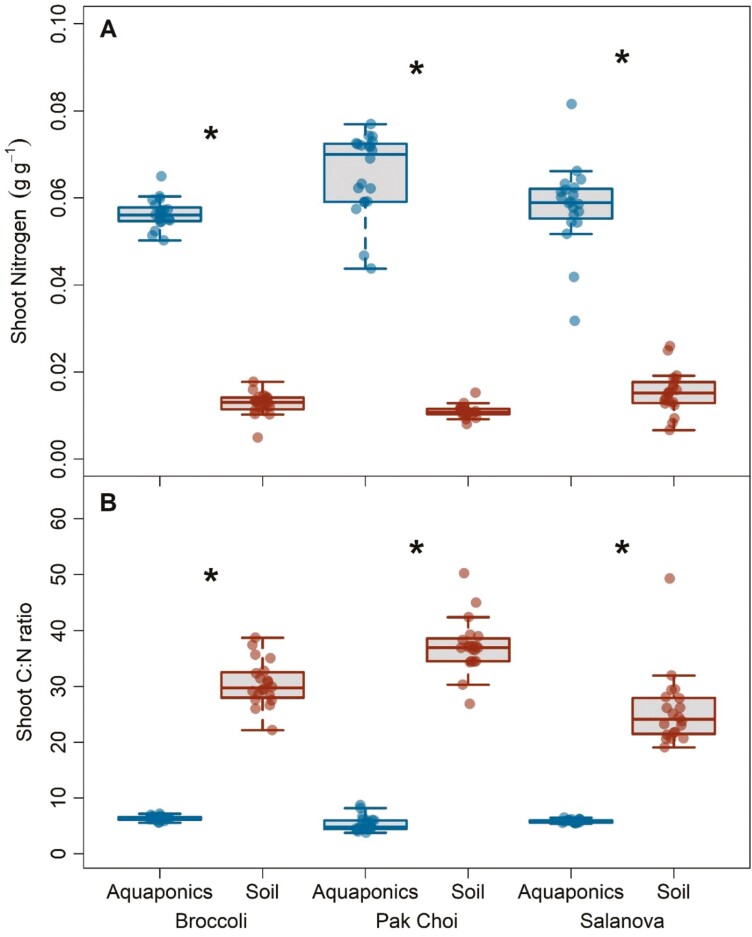
Boxplots of (A) shoot nitrogen content and (B) shoot C:N ratio for three leafy green species grown in aquaponics and soil treatments. Asterisks represent significant differences between growth treatments for a given species. Each box represents the interquartile range, the line is the median, and the whiskers extend to the lower and upper limits of the data.

Tissue nitrogen content of both shoots and roots was higher in aquaponics across all three species. Shoot *N* content increased by 367%, 491%, and 287% in broccoli, pak choi, and salanova respectively (Fig. 4A, all *P* < 0.001). Consequently, the C:N ratio of shoots broadly decreased across all species in aquaponics (Fig. 4B, all *P* < 0.001). Root *N* content also increased for each species in aquaponics by 167%, 100%, and 444%, respectively for broccoli, pak choi, and salanova (Supplementary Table S1, all *P* < 0.001). Similarly, the C:N ratio of roots decreased across all species in aquaponics (Supplementary Table S1, all *P* < 0.001). Bulk shoot δ^13^C was significantly lower in broccoli and pak choi plants grown in aquaponics compared to soil (*P* = 0.004 and <0.001, respectively), while values were similar for salanova between treatments (Fig. 3B).

Acclimatory changes in stomatal traits between aquaponics and soil treatments varied by species (Supplementary Table S1). The stomatal density of aquaponics-grown broccoli and salanova was lower by 31% and 28%, respectively, compared to soil-grown plants (Fig. 4C, *P* < 0.001). Stomatal density in leaves of pak choi did not significantly change between treatments. Stomatal density (SD) for broccoli and salanova did not change through time in aquaponics, however, SD in both species did increase when grown in soil between weeks 1 and 2 (SD * week, *P* < 0.001 and *P* = 0.014, respectively). Stomatal density in pak choi in aquaponics increased from week 1 to 4 (SD * week, *P* = 0.024) while pak choi in soil increased from week 1 to 2 (SD * week, *P* < 0.001). No changes were detected in stomatal size for pak choi and salanova, while stomatal size for broccoli increased by 16% in aquaponics compared to soil treatments (*P* < 0.001).

### Bivariate relationships between functional traits

**Figure 5.**
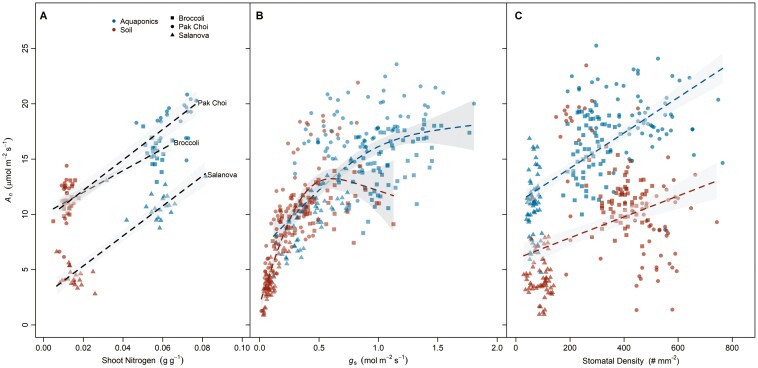
Relationships between light saturated photosynthesis (*A*_*n*_) and (A) shoot nitrogen content, (B) stomatal conductance, and (C) stomatal density. For each treatment, dashed lines represent significant linear or generalized additive model fits and gray shaded areas are 95 % confidence intervals for the mean.

A positive relationship between *A*_*n*_ and shoot nitrogen (*N*) existed across all plants (*P* < 0.001, *R*^2^  _marginal_ = 0.37, *R*^2^  _conditional_ = 0.90). However, the *A*_*n*_—*N* relationship was not apparent within treatments (aquaponics nor soil), due to a large amount of variation across species. Across treatments (Fig. 5A), increases in shoot nitrogen were positively correlated with increases in *A*_*n*_ for broccoli (*P* < 0.001, *R*^2^ = 0.79), pak choi (*P* < 0.001, *R*^2^ = 0.78), and salanova (*P* < 0.001, *R*^2^ = 0.80). Light-saturated photosynthesis increased with higher *g*_*s*_ across species for both treatments until *A*_*n*_ plateaued at larger rates of *g*_*s*_ (*P* < 0.001, deviance explained = 84.1%). Importantly, three-fold increases in *g*_*s*_ in some aquaponics plants led to much higher rates of *A*_n_ (*P* < 0.001, Fig. 5B). Light-saturated photosynthesis rates were also positively related to stomatal density despite large amounts of variation between species (*P* = 0.001, *R*^2^  _marginal_ = 0.25, *R*^2^  _conditional_ = 0.81). The slopes of the *A*_*n*_—SD relationship also differed across treatments, as *A*_*n*_ aquaponics plants responded more strongly to changes in SD (*P* = 0.001, Fig. 5C). Intrinsic water use efficiency was not related to SD for either treatment.

## Discussion

In a novel aquaponics experiment, we tested the capacity of three leafy green species to physiologically acclimate to unrestricted water availability and consistent nitrogen supply from fish efflux compared to growth in limited soil volume. We found biomass production, rates of photosynthesis, and stomatal conductance to be higher in aquaponics, while intrinsic water use efficiency was lower in aquaponics for all species. Although the relationship between photosynthetic rates (*A*_*n*_) and either foliar nitrogen (*N*) or stomatal conductance (*g*_s_) is well understood, our results show the potential of plants to acclimate key photosynthetic traits to optimize physiology in a unique growth system. Our findings offer mechanistic insight into the future of sustainable farming that utilizes aquaponics to produce non-staple crops that meaningfully contribute to feed insecurity.

### Changes in growth and biomass partitioning in aquaponics

Full biomass harvests showed that total biomass production of leafy green crops vastly increased in aquaponics compared to unfertilized soil. Utilizing aquaponics systems, as a substitute or complement to soil-based agriculture, contributes to stable food production while also increasing efficiency of water usage and reduction in reliance on fertilizer. For example, compared to conventional agricultural methods paired with pond-based or lake-based aquaculture, aquaponics was found to conserve water usage by greater than 75% (Cohen *et al.* 2018). Surprisingly, comparisons of biomass growth for key crop species between aquaponics systems and soil are largely unrepresented in the aquaponics literature. Aquaponic crop production is more commonly compared to hydroponic systems, with aquaponics systems producing higher yields (Johnson *et al.* 2017; Lennard and Ward 2019). Many species grow well in aquaponics systems due to their nutritional needs being supplied by the fish efflux (Krastanova *et al.* 2022), however, plant growth in aquaponics is linked to tradeoffs associated with maintaining fish and plant needs simultaneously, including pH, temperature, and nutrient compositions (Delaide *et al.* 2019). Although our study does indicate increased biomass production is possible in aquaponics, the magnitude of the increase is very likely overestimated compared to soil-based agriculture with fertilizer regimes. Nonetheless, Ranawade Tidke and Kate (2017) found the biomass production of spinach to be the highest when grown in an aquaponics system compared to both a hydroponics system and a traditional soil method with several fertilizers.

Two of the species, broccoli and salanova, also exhibited decreased root:shoot ratios when grown in aquaponics. Optimal partitioning theory describes changes in biomass allocation to environmental factors (e.g. water, light, and CO_2_) by correlating belowground deficiencies with higher root growth and above-ground deficiencies with an increase in aboveground biomass (Reynolds and Thornley 1982; Johnson and Thornley 1987; Poorter *et al.* 2012; Ledo *et al.* 2018). For example, shifts in biomass allocation are often linked to water stress. Increases in belowground biomass as a response to drought conditions can result in increases in root:shoot ratios (Xu *et al.* 2015a). In this experiment, without belowground stressors for water or nutrients driving an increased carbon sink in roots for resource uptake, aquaponic broccoli, and salanova partitioned growth more towards aboveground biomass. Simply, aquaponics plants of these species allocated more resources into aboveground biomass without concurrent increased investment in root tissue.

### Acclimation of gas exchange in aquaponics

Photosynthetic rate and stomatal conductance significantly increased in all three leafy green species when grown in aquaponics. Our results support the stomatal optimality theory that stomata regulate functions to balance carbon uptake with the penalties of open stomata (Buckley and Schymanski 2014), albeit with a phenomenon that is less often observed. With a continuous supply of water, aquaponics plants acclimated stomatal behavior by maintaining high rates of *g*_s_ because the consequences of transpiration were negligible compared to plants grown in soil. This acclimation of stomatal behavior in aquaponics plants contributed to concurrent higher rates of *A*_*n*_. As instantaneous photosynthetic rates are a function of stomatal opening and the biochemical parameters (*V*_cmax_ and *J*_max_) that regulate photosynthetic capacity (Wang *et al.* 2020), further research on the capacity of N-rich fish efflux to enhance rates of Rubisco carboxylation and/or electron transport for RuBP regeneration in aquaponics plants are still needed.

Importantly, stomatal responses can respond slower than photosynthetic responses to environmental factors, often limiting the capacity of *A*_*n*_ (Lawson and Blatt 2014; Lawson and Vialet-Chabrand 2019). Here, the lag between stomatal responses to external factors was inconsequential for plants grown in the controlled aquaponics environment, allowing for optimization of carbon gain. Thus, the larger rates of *g*_s_ exhibited across species in aquaponics did result in some apparent degree of saturation of *A*_*n*_ at a given light and temperature environment. Future studies in aquaponics should prioritize optimizing light regimes and growth temperatures to test if crop plants can further optimize the acclimation of gas exchange.

Our study found *N* supply to be significantly higher in aboveground and belowground tissues in plants grown in aquaponics. Crop plants, like the ones used in this study, are sensitive to changes in *N* supply and *N* limitation can drive changes in leaf traits and *N* investment to maintain photosynthetic capacity (Vos Van Der Putten and Birch 2005). For example, leaves rely on sufficient *N* intake to produce essential photosynthetic proteins like Rubisco, creating an extensive *N* sink to support photosynthesis and subsequent growth (Gastal *et al.* 2015; Evans and Clarke 2019). Here, higher rates of *A*_*n*_ for all three species in aquaponics were correlated with increased leaf nitrogen content. Aquaponics plants simply had access to more N to allocate to photosynthetic machinery, without the need to invest in higher root production. The access to a larger pool of *N* likely allowed the upregulated *A*_*n*_ rates to meet the sink demands of aboveground organs (Zhang *et al.* 2015; Tegeder and Masclaux-Daubresse 2018). The access to a continuous pool of *N* also resulted in a consistently lower C:N ratio of both above- and belowground tissues in aquaponics plants. With lower C:N ratios, plants can prioritize growth in nitrogen-sufficient environments compared to the need to prioritize nitrogen use efficiency (Zhang *et al.* 2019). Further work should determine the degree to which typical fertilization regimes in soil-based agriculture close the observed gap in plant nitrogen economy with aquaponics.

### Water-use “”inefficiency” in aquaponics plants

As hypothesized, one of the strongest acclimatory responses in aquaponics plants was a functional shift towards water-use inefficiency in leaf gas exchange. Stomatal behavior defines a plant’s water use efficiency, as the pore acts as the resistant force towards atmosphere flux and internal stimuli interact with CO_2_ flux and transpiration (Lawson and Blatt 2014; de Santana *et al.* 2015; Zhao *et al.* 2020). We detected aspects of these shifts in both adjustments in physiological behavior and anatomical traits. Instantaneous transpiration efficiency was lower in all three leafy green species when grown in aquaponics. For broccoli and pak choi, the removal of water limitation in aquaponics plants also drove decreases in water-use efficiency across the leaf life span (lower foliar δ^13^C content), revealing likely sustained higher uptake of CO_2_ and decreased water-use efficiency across the experiment.

Contrary to our hypothesis, stomata density (SD) decreased in aquaponics. Water vapor losses from leaves are functions of the size, density, and distributions of stomata (Franks and Beerling 2009; Dow Berry and Bergmann 2014; Fanourakis *et al.* 2015; Devi and Reddy 2018). Our results relate to evidence highlighted by Lawson and Blatt (2014) that decreased SD can be compensated for with an increase in stomatal aperture. Here, aquaponic plants increased *g*_s_ by leaving stomata pores more open, while also investing less into creating more stomata on newly created leaves. Our study uniquely focused on an environment without water stress and consistent light and CO_2_ levels that is seldom found in literature as investigations of stomatal traits often involve responses to drought, light regimes, or altered CO_2_ levels (Bertolino Caine and Gray 2019). For example, changes to stomatal aperture size, without acclimation of SD, was detected in *Arabidopsis* plants in response to drought (Doheny-Adams *et al.* 2012). Additionally, Xu and Zhou (2008) found a positive correlation between SD and water-use efficiency, *g*_s_, and *A*_*n*_ in false wheatgrass experiencing drought conditions. In contrast to these drought studies, the ability of aquaponics plants to maintain consistent levels of water-use “inefficiency“ via high *g*_s_, allowed concurrent upregulation of *A*_*n*_ and down regulation of stomatal production. Experiments in both aquaponics and hydroponics systems should continue to prioritize understanding how acclimation of plant water-use strategies can be harnessed to optimize crop production.

### Acclimation potential of aquaponics plants

Plants are known to acclimate to a variety of changing environmental conditions such as cold temperatures (Hassan *et al.* 2021; Juurakko *et al.* 2021a, b), diffused light (Li *et al.* 2014; Liu and Su 2016), high salinity (Hossain *et al.* 2017; Pandolfi *et al.* 2017), and drought (Pandey and Shukla 2015; Ahmad *et al.* 2016; Menezes-Silva *et al.* 2017). Currently, evidence of plant acclimation in hydroponics growth environments compared to soil-based growth is mostly absent in the literature. Overall, these leafy green species were capable of adjustments to the aquaponic growth environment for key processes that regulate photosynthesis and growth by the time the initial cohort of leaves was fully formed. Here, gas exchange variables of all species had acclimated to aquaponics when the first cohort of leaves was measured, and stomatal anatomy had acclimated by the second cohort of leaves. As future food security concerns become a driving force in agricultural research, the acclimation potential exhibited by the hydroponically grown crops in this study should be more broadly investigated to improve precision farming.

### Summary

Connections between decreasing human health from malnourishment resulting from climate change continue to rise as concerns grow for future food and freshwater availability, soil health, and biodiversity (Mcmichael 2013). Our experimental findings that functional traits of different leafy green species all acclimated to enhance photosynthesis in aquaponics provide evidence that aquaponics has the capacity to improve production on large and small scales and that aquaponics systems could become a key contributor to global food security as the system is unaffected by urbanization, environmental degradation, and climate change (Ghandar *et al.* 2021). Although not part of the study, dietary deficiencies can be combatted through increased consumption of fish which are high in protein and contain vitamin A, B, and D along with essential micronutrients like calcium, iron, and zinc (Béné *et al.* 2015). Thus, the capacity of aquaponics to support sustainable vegetarian and pescetarian style diets could combat health concerns, climate change complications like drought, and food security risks by relying on the improved vegetable production capacities of aquaponics systems (Springmann *et al.* 2016). Higher investments in aquaponics growing systems could meaningfully contribute to the supply of crucial greens, herbs, vegetables, and fish needed to support the growing human population in an agriculture world plagued by global change (Shreejana *et al.* 2022).

## Supplementary Material

plaf005_suppl_Supplementary_Materials

## Data Availability

The data that support the findings of this study are openly available at https://figshare.com/projects/Aquaponics_Physiology_dataset_Nicholes_et_al_2025_AoB_Plants/233471.

## References

[CIT0001] Abdalla M, Ahmed MA, Cai G et al Stomatal closure during water deficit is controlled by below-ground hydraulics. Ann Bot (Lond) 2022;129:161–70. https://doi.org/10.1093/aob/mcab141 PMC8796668 34871349

[CIT0002] Ahanger MA et al Plant growth under drought stress: significance of mineral nutrients. Water Stress and Crop Plants: A sustainable Approach 2016;2:649–68. https://doi.org/10.1002/9781119054450.ch37

[CIT0003] Ahmad N, Malagoli M, Wirtz M et al Drought stress in maize causes differential acclimation responses of glutathione and sulfur metabolism in leaves and roots. BMC Plant Biol 2016;16:1–15. https://doi.org/10.1186/S12870-016-0940-Z 27829370 PMC5103438

[CIT0004] AlShrouf A. Hydroponics, aeroponic and aquaponic as compared with conventional farming. AASRJETS 2017;27:247–55. https://asrjetsjournal.org/index.php/American_Scientific_Journal/article/view/2543 Accessed 24 October 2022.

[CIT0005] Asseng S, Guarin JR, Raman M et al Wheat yield potential in controlled-environment vertical farms, . Proc Natl Acad Sci USA 2020;117:19131–5. https://doi.org/10.1073/pnas.2002655117 32719119 PMC7430987

[CIT0006] Bebber DP, Holmes T, Gurr SJ. The global spread of crop pests and pathogens. Glob Ecol Biogeogr 2014;23:1398–407. https://doi.org/10.1111/geb.12214

[CIT0007] Béné C, Barange M, Subasinghe R et al Feeding 9 billion by 2050—putting fish back on the menu. Food Security 2015;7:261–74. https://doi.org/10.1007/s12571-015-0427-z

[CIT0008] Bertolino LT, Caine RS, Gray JE. Impact of stomatal density and morphology on water-use efficiency in a changing world. Front Plant Sci 2019;10:225. https://doi.org/10.3389/fpls.2019.00225 30894867 PMC6414756

[CIT0009] Bisbis MB, Gruda N, Blanke M. Potential impacts of climate change on vegetable production and product quality—a review. J Clean Prod 2018;170:1602–20. https://doi.org/10.1016/j.jclepro.2017.09.224

[CIT0010] Bouzid M, He F, Schmitz G et al Arabidopsis species deploy distinct strategies to cope with drought stress. Ann Bot (Lond) 2019;124:27–40. https://doi.org/10.1093/aob/mcy237 PMC6676377 30668651

[CIT0011] Boyer JS, Byrne P, Cassman KG et al The U.S. drought of 2012 in perspective: a call to action. Glob Food Secur 2013;2:139–43. https://doi.org/10.1016/j.gfs.2013.08.002

[CIT0012] Buckley TN, Schymanski SJ. Stomatal optimisation in relation to atmospheric CO_2_. New Phytol 2014;201:372–7. http://www.jstor.org/stable/newphytologist.201.2.372. 24124922 10.1111/nph.12552

[CIT0013] Cairns JE, Hellin J, Sonder K et al Adapting maize production to climate change in sub-Saharan Africa. Food Secur 2013;5:345–60. https://doi.org/10.1007/s12571-013-0256-x

[CIT0014] Carminati A, Javaux M. Soil rather than xylem vulnerability controls stomatal response to drought. Trends Plant Sci 2020;25:868–80. https://doi.org/10.1016/j.tplants.2020.04.003 32376085

[CIT0015] Cemek B et al Effects of evapotranspiration and soil salinity on some growth parameters and yield of lettuce (*Lactuca sativa* var. crispa). *Zemdirbyste-Agriculture* 2011;98:139–48.

[CIT0016] Chouchane H, Krol MS, Hoekstra AY. Expected increase in staple crop imports in water-scarce countries in 2050. Water Research X 2018;1:100001. https://doi.org/10.1016/j.wroa.2018.09.001 31193997 PMC6549899

[CIT0017] Clark M, Tilman D. Comparative analysis of environmental impacts of agricultural production systems, agricultural input efficiency, and food choice. Environ Res Lett 2017;12:064016. https://doi.org/10.1088/1748-9326/aa6cd5

[CIT0018] Cleland J. World population growth; past, present and future. Environ Resource Econ 2013;55:543–54. https://doi.org/10.1007/s10640-013-9675-6

[CIT0019] Cohen A, Malone S, Morris Z et al Combined fish and lettuce cultivation: an aquaponics life cycle assessment. Procedia CIRP 2018;69:551–6. https://doi.org/10.1016/j.procir.2017.11.029

[CIT0020] Cole MB, Augustin MA, Robertson MJ et al PERSPECTIVE The science of food security. npj Sci Food 2018;2:14. https://doi.org/10.1038/s41538-018-0021-9 31304264 PMC6550266

[CIT0021] Condon AG. Drying times: plant traits to improve crop water use efficiency and yield. J Exp Bot 2020;71:2239–52. https://doi.org/10.1093/jxb/eraa002 31912130

[CIT0022] Conti L. The A-B-A of floral transition: the to do list for perfect escape. Mole Plant 2019;12:289–91. https://doi.org/10.1016/j.molp.2019.02.002 30790664

[CIT0023] Dalin C, Wada Y, Kastner T et al Groundwater depletion embedded in international food trade NASA Public Access. Nature 2017;543:700–4. https://doi.org/10.1038/nature21403 28358074 PMC7427584

[CIT0024] Davidson EA, Suddick EC, Rice CW et al More food, low pollution (Mo Fo Lo Po): a grand challenge for the 21st century. J Environ Qual 2015;44:305–11. https://doi.org/10.2134/jeq2015.02.0078 26023950

[CIT0025] Delaide B, Monsees H, Gross A, Goddek S Aerobic and anaerobic treatments for aquaponic sludge reduction and mineralisation. Aquaponics Food Prod Systems 2019;1: 247–66.

[CIT0026] de Santana TA, Oliveira PS, Silva LD et al Water use efficiency and consumption in different Brazilian genotypes of *Jatropha curcas* L. subjected to soil water deficit. Biomass Bioenergy 2015;75:119–25. https://doi.org/10.1016/j.biombioe.2015.02.008

[CIT0027] Devi MJ, Reddy VR. Transpiration response of cotton to vapor pressure deficit and its relationship with stomatal traits. Front Plant Sci 2018;9:1572. https://doi.org/10.3389/FPLS.2018.01572 30420866 PMC6218332

[CIT0028] Dietz KJ, Zörb C, Geilfus CM. Drought and crop yield. Plant Biol 2021, 23:881–93. https://doi.org/10.1111/plb.13304 34396653

[CIT0029] Doheny-Adams T, Hunt L, Franks PJ et al Genetic manipulation of stomatal density influences stomatal size, plant growth and tolerance to restricted water supply across a growth carbon dioxide gradient. Philos Trans R Soc B, Biol Sci 2012;367:547–55. https://doi.org/10.1098/rstb.2011.0272 PMC3248714 22232766

[CIT0030] Dow GJ, Berry JA, Bergmann DC. The physiological importance of developmental mechanisms that enforce proper stomatal spacing in *Arabidopsis thaliana*. New Phytol 2014;201:1205–17. 24206523 10.1111/nph.12586

[CIT0031] Dror I, Yaron B, Berkowitz B. The human impact on all soil-forming factors during the anthropocene. 2021;2:11–9. 10.1021/acsenvironau.1c00010 PMC10114744 37101758

[CIT0032] Evans JR. Photosynthesis and nitrogen relationships in leaves of C3 plants. Oecologia 1989;78:9–19. https://doi.org/10.1007/BF00377192 28311896

[CIT0033] Evans JR, Clarke VC. The nitrogen cost of photosynthesis. J Exp Bot 2019;70:7–15. https://doi.org/10.1093/jxb/ery366 30357381

[CIT0034] Evans JR, Seemann JR. The allocation of protein nitrogen in the photosynthetic apparatus: costs, consequences, and control. Photosynthesis 1989;8:183–205.

[CIT0035] Fahad S, Bajwa, Ali A et al Crop production under drought and heat stress: plant responses and management options. Front Plant Sci 2017;8:1147. https://doi.org/10.3389/fpls.2017.01147 28706531 PMC5489704

[CIT0036] Fanourakis D, Giday H, Milla R et al Pore size regulates operating stomatal conductance, while stomatal densities drive the partitioning of conductance between leaf sides. Ann Bot (Lond) 2015;115:555–65. https://doi.org/10.1093/aob/mcu247 PMC4343285 25538116

[CIT0037] Fox J, Weisberg S. 2018. An R Companion to Applied Regression. Thousand Oaks, CA, USA: Sage publications.

[CIT0038] Franks PJ, Beerling DJ. Maximum leaf conductance driven by CO_2_ effects on stomatal size and density over geologic time. Proc Natl Acad Sci USA 2009;106:10343–7. https://doi.org/10.1073/pnas.0904209106 19506250 PMC2693183

[CIT0039] Gastal, F. et al Quantifying crop responses to nitrogen and avenues to improve nitrogen-use efficiency. In Sadras VO and Calderini DE (eds.), Crop Physiology: Application for Genetic Improvement and Agronomy, 2nd Edn. San Diego, CA, USA: Elsevier Inc., 2015, 161–206. Available at: https://doi.org/10.1016/B978-0-12-417104-6.00008-X

[CIT0040] Gerten D, Heck V, Jägermeyr J et al Feeding ten billion people is possible within four terrestrial planetary boundaries. Nat Sustainability 2020;3:200–8. https://doi.org/10.1038/s41893-019-0465-1

[CIT0041] Ghandar A et al A decision support system for urban agriculture using digital twin: a case study with aquaponics. IEEE 2021;9:35691–708. https://doi.org/10.1109/ACCESS.2021.3061722

[CIT0042] Grafton RQ, Williams J, Perry CJ et al The paradox of irrigation efficiency. Science 2018;361:748–50. https://doi.org/10.1126/science.aat9314 30139857

[CIT0043] Grossiord C, Buckley TN, Cernusak LA et al Plant responses to rising vapor pressure deficit. New Phytol 2020;226:1550–66. https://doi.org/10.1111/nph.16485 32064613

[CIT0044] Grzebisz W, Gransee A, Szczepaniak W et al The effects of potassium fertilization on water-use efficiency in crop plants. J Plant Nutr Soil Sci 2013;176:355–74. https://doi.org/10.1002/jpln.201200287

[CIT0045] Hanes C, Rhode PW. Harvests and financial crises in gold standard America. *J Econ Hist* 2013;73:201–46. https://doi.org/10.1017/S0022050713000077

[CIT0046] Hassan MA, Xiang C, Farooq M et al Cold stress in wheat: plant acclimation responses and management strategies. Front Plant Sci 2021;12:1234. https://doi.org/10.3389/FPLS.2021.676884/BIBTEX PMC8299469 34305976

[CIT0047] Hossain MS, ElSayed AI, Moore M et al Redox and reactive oxygen species network in acclimation for salinity tolerance in sugar beet. J Exp Bot 2017;68:1283–98. https://doi.org/10.1093/jxb/erx019 28338762 PMC5441856

[CIT0048] Houlton BZ, Almaraz M, Aneja V et al A world of cobenefits: solving the global nitrogen challenge. Earths Future 2019;7:865–72. https://doi.org/10.1029/2019ef001222 PMC6733275 31501769

[CIT0049] Janni YD, Jadhav DB. Aquaponics uses, cultivation and beneficial effects. Just Agricult 2022;2:1–7.

[CIT0050] Johnson G et al Evaluation of lettuce between spring water, hydroponic, and flow-through aquaponic systems. Int J .Veg Sci 2017;23:456–70. https://doi.org/10.1080/19315260.2017.1319888

[CIT0051] Johnson IR, Thornley JHM. A model of shoot: root partitioning with optimal growth. Ann Bot (Lond) 1987;60:133–42. https://doi.org/10.1093/oxfordjournals.aob.a087429

[CIT0052] Jones DL, Cross P, Withers PJA et al REVIEW: Nutrient stripping: the global disparity between food security and soil nutrient stocks. J Appl Ecol 2013;50:851–62. https://doi.org/10.1111/1365-2664.12089

[CIT0053] Juurakko CL, Bredow M, Nakayama T et al The *Brachypodium distachyon* cold-acclimated plasma membrane proteome is primed for stress resistance. G3 Genes|Genomes|Genetics 2021a;11, jkab198. https://doi.org/10.1093/G3JOURNAL/JKAB198 PMC8661430 34544140

[CIT0054] Juurakko CL, diCenzo GC, Walker VK. Cold acclimation and prospects for cold-resilient crops. Plant Stress 2021b;2:100028. https://doi.org/10.1016/j.stress.2021.100028

[CIT0055] Krastanova M, Sirakov I, Ivanova-Kirilova S et al Aquaponic systems: biological and technological parameters. Biotechnol Biotechnol Equip 2022;36:305–16. https://doi.org/10.1080/13102818.2022.2074892

[CIT0056] Kumar K, Gambhir G, Dass A et al Genetically modified crops: current status and future prospects. Planta 2020;251:1–27. https://doi.org/10.1007/s00425-020-03372-8 32236850

[CIT0057] Ladics GS, Bartholomaeus A, Bregitzer P et al Genetic basis and detection of unintended effects in genetically modified crop plants. Transgenic Res 2015;24:587–603. https://doi.org/10.1007/s11248-015-9867-7 25716164 PMC4504983

[CIT0058] Lawson T, Blatt MR. Topical review on stomata and water use efficiency stomatal size, speed, and responsiveness impact on photosynthesis and water use efficiency. Plant Physiol 2014;164:1556–70. https://doi.org/10.1104/pp.114.237107 24578506 PMC3982722

[CIT0059] Lawson T, Vialet-Chabrand S. Speedy stomata, photosynthesis and plant water use efficiency. New Phytol 2019;221:93–8. https://doi.org/10.1111/NPH.15330 29987878

[CIT0060] Ledo A et al Tree size and climatic water deficit control root to shoot ratio in individual trees globally. New Phytol 2018;217:8–11. https://www.jstor.org/stable/90016341 29058312 10.1111/nph.14863

[CIT0061] Leng G, Hall J. Crop yield sensitivity of global major agricultural countries to droughts and the projected changes in the future. Sci Total Environ 2019;654:811–21. https://doi.org/10.1016/j.scitotenv.2018.10.434 30448671 PMC6341212

[CIT0062] Lennard W, Ward J. A comparison of plant growth rates between an NFT hydroponic system and an NFT aquaponic system. Horticulturae 2019;5:27. https://doi.org/10.3390/horticulturae5020027

[CIT0063] Lenth RV Emmeans: Estimated marginal means, aka least-squares means. *R* *package version 1.7.2*. 2022.

[CIT0064] Li T, Heuvelink E, Dueck TA et al Enhancement of crop photosynthesis by diffuse light: quantifying the contributing factors. Ann Bot (Lond) 2014;114:145–56. https://doi.org/10.1093/aob/mcu071 PMC4071095 24782436

[CIT0065] Liu W, Su J. Effects of light acclimation on shoot morphology, structure, and biomass allocation of two Taxus species in southwestern China. Sci Rep 2016;6:1–9. https://doi.org/10.1038/srep35384 27734944 PMC5062112

[CIT0066] Long SP, Marshall-Colon A, Zhu XG. Meeting the global food demand of the future by engineering crop photosynthesis and yield potential. Cell 2015;161:56–66. https://doi.org/10.1016/j.cell.2015.03.019 25815985

[CIT0067] Martignago D, Rico-Medina A, Blasco-Escámez D et al Drought resistance by engineering plant tissue-specific responses. Front Plant Sci 2020;10:1676. https://doi.org/10.3389/fpls.2019.01676. Frontiers Media S.A. 32038670 PMC6987726

[CIT0068] Mcmichael AJ. Globalization, climate change, and human health. N Engl J Med 2013;368:1335–43. https://doi.org/10.1056/NEJMra1109341 23550671

[CIT0069] Menezes-Silva PE, Sanglard LMVP, Ávila RT et al Photosynthetic and metabolic acclimation to repeated drought events play key roles in drought tolerance in coffee. J Exp Bot 2017;68:4309–22. https://doi.org/10.1093/jxb/erx211 28922767

[CIT0070] Mote S, Rivas J, Kalnay E. A novel approach to carrying capacity: from a priori prescription to a posteriori derivation based on underlying mechanisms and dynamics. Annu Rev Earth Planet Sci 2020;48:657–83. https://doi.org/10.1146/annurev-earth-053018-060428

[CIT0071] Mueller ND, Gerber JS, Johnston M et al Closing yield gaps through nutrient and water management. Nature 2012;490:254–7. https://doi.org/10.1038/nature11420 22932270

[CIT0072] Nabout JC, Magalhães MR, de Amorim Gomes MA et al The impact of global climate change on the geographic distribution and sustainable harvest of *Hancornia speciosa* Gomes (Apocynaceae) in Brazil. Environ Manage 2016;57:814–21. https://doi.org/10.1007/s00267-016-0659-5 26796699

[CIT0073] Nakagawa S, Schielzeth H. A general and simple method for obtaining R2 from generalized linear mixed-effects models. Methods Ecol Evol 2013;4:133–42. https://doi.org/10.1111/J.2041-210X.2012.00261.X

[CIT0074] Ozeki K, Miyazawa Y, Sugiura D. Rapid stomatal closure contributes to higher water use efficiency in major C 4 compared to C 3 Poaceae crops. Plant Physiol 2022;189:188–203. https://doi.org/10.1093/plphys/kiac040 35134220 PMC9070804

[CIT0075] Palm HW, Knaus U, Appelbaum S et al Towards commercial aquaponics: a review of systems, designs, scales and nomenclature. Aquac Int 2018;26:813–42. https://doi.org/10.1007/s10499-018-0249-z

[CIT0076] Pandey V, Shukla A. Acclimation and tolerance strategies of rice under drought stress. Rice Sci 2015;22:147–61. https://doi.org/10.1016/j.rsci.2015.04.001

[CIT0077] Pandolfi C, Bazihizina N, Giordano C et al Salt acclimation process: a comparison between a sensitive and a tolerant *Olea europaea* cultivar. Tree Physiol 2017;37:380–8. https://doi.org/10.1093/treephys/tpw127 28338715

[CIT0078] Paulot F, Jacob DJ. Hidden cost of U.S. agricultural exports: particulate matter from ammonia emissions. Environ Sci Technol 2014;48:903–8. https://doi.org/10.1021/es4034793 24370064

[CIT0079] Peña-Gallardo M, Vicente-Serrano SM, Domínguez-Castro F et al Effectiveness of drought indices in identifying impacts on major crops across the USA. Clim Res 2018;75:221–40. https://doi.org/10.3354/cr01519

[CIT0080] Perrone D, Jasechko S. Dry groundwater wells in the western United States. Environ Res Lett 2017;12:104002. https://doi.org/10.1088/1748-9326/aa8ac0

[CIT0081] Philip Robertson G et al Nitrogen–climate interactions in US agriculture. *Biogeochemistry* 2013;114:41–70. https://doi.org/10.1007/s10533-012-9802-4

[CIT0082] Poorter H et al Biomass allocation to leaves, stems and roots: meta-analyses of interspecific variation and environmental control. New Phytol 2012;193:30–50 https://doi.org/10.1111/J.1469-8137.2011.03952.X 22085245

[CIT0083] R Development Core Team. R: A Language and Environment for Statistical Computing. Vienna, Austria; 2017.

[CIT0084] Ramankutty N, Mehrabi Z, Waha K et al Trends in global agricultural land use: implications for environmental health and food security. Annu Rev Plant Biol 2018;69:789–815. https://doi.org/10.1146/annurev-arplant-042817-040256 29489395

[CIT0085] Ranawade PS, Tidke SD, Kate AK. Comparative cultivation and biochemical analysis of *Spinacia oleraceae* grown in aquaponics, hydroponics and field conditions. Int J Curr Microbiol Appl Sci 2017;6:1007–13. https://doi.org/10.20546/ijcmas.2017.604.125

[CIT0086] Ray DK, Mueller ND, West PC et al Yield trends are insufficient to double global crop production by 2050. PLoS One 2013;8:e66428. https://doi.org/10.1371/journal.pone.0066428 23840465 PMC3686737

[CIT0087] Reynolds JF, Thornley JHM. A shoot: root partitioning model. Ann Bot (Lond) 1982;49:585–97. Available at: https://about.jstor.org/terms

[CIT0088] Richards RA. Selectable traits to increase crop photosynthesis and yield of grain crops. J Exp Bot 2000;51 Spec No:447–58. https://doi.org/10.1093/jexbot/51.suppl_1.447 10938853

[CIT0089] Richards RA. Is nitrogen a key determinant of water transport and photosynthesis in higher plants upon drought stress? Front Plant Sci 2018;9:1143. https://doi.org/10.3389/fpls.2018.01143 30186291 PMC6113670

[CIT0090] Rigden AJ, Mueller ND, Holbrook NM et al Combined influence of soil moisture and atmospheric evaporative demand is important for accurately predicting US maize yields. Nature Food 2020;1:127–33. https://doi.org/10.1038/s43016-020-0028-7. 37127990

[CIT0091] Roberts L. 9 Billion? Science 2011;333:540–3. https://doi.org/10.1126/science.333.6042.540 21798924

[CIT0092] Rockström J, Williams J, Daily G et al Sustainable intensification of agriculture for human prosperity and global sustainability. Ambio 2017;46:4–17. https://doi.org/10.1007/s13280-016-0793-6 27405653 PMC5226894

[CIT0093] Rosa-Schleich J, Loos J, Mußhoff O et al Ecological-economic trade-offs of diversified farming systems—a review. Ecolog Econ 2019;160:251–63. https://doi.org/10.1016/j.ecolecon.2019.03.002

[CIT0094] Santoni M, Ferretti L, Migliorini P et al A review of scientific research on biodynamic agriculture. Org Agr 2022;12:373–96. https://doi.org/10.1007/s13165-022-00394-2.

[CIT0095] Scheelbeek PF. et al Effect of environmental change on vegetable and legumes yields and nutriional quality. *Proceedings of the National Academy of Sciences of the United States of America* 2018;115:6804–9. https://doi.org/10.1073/pnas.1800442115. 29891659 PMC6042093

[CIT0096] Shah AN, Tanveer M, Shahzad B et al Soil compaction effects on soil health and cropproductivity: an overview. Environ Sci Pollut Res 2017;24:10056–67. https://doi.org/10.1007/s11356-017-8421-y 28108925

[CIT0097] Shreejana KC et al Aquaponics a modern approach for integrated farming and wise utilization of components for sustainability of food security: a review. Arch Agric Environ Sci 2022;7:121–6. https://doi.org/10.26832/24566632.2022.0701017

[CIT0098] Siegel FR. Impact of global warming/climate change on food security 2020. In: Siegel FR (ed.), The Earth’s Human Carrying Capacity: Limitations Assessed, Solutions Proposed, Cham, Switzerland: Springer International Publishing. 2021: 1–5. https://doi.org/10.1007/978-3-030-73476-3_1

[CIT0099] Springmann M, Godfray HCJ, Rayner M et al Analysis and valuation of the health and climate change cobenefits of dietary change. Proc Natl Acad Sci USA 2016;113:4146–51. https://doi.org/10.1073/pnas.1523119113 27001851 PMC4839446

[CIT0100] Suh J. Towards sustainable agricultural stewardship: evolution and future directions of the permaculture concept. Environ Values 2014;23:75–98. https://doi.org/10.3197/096327114x13851122269089

[CIT0101] Tai APK, Martin MV, Heald CL. Threat to future global food security from climate change and ozone air pollution. Nat Clim Change 2014;4:817–21. https://doi.org/10.1038/nclimate2317

[CIT0102] Tegeder M, Masclaux-Daubresse C. Source and sink mechanisms of nitrogen transport and use. New Phytol 2018;217:35–53. https://doi.org/10.1111/NPH.14876 29120059

[CIT0103] Verdoliva SG, Gwyn-Jones D, Detheridge A et al Controlled comparisons between soil and hydroponic systems reveal increased water use efficiency and higher lycopene and β-carotene contents in hydroponically grown tomatoes. Sci Horticult 2021;279:109896. https://doi.org/10.1016/j.scienta.2021.109896 PMC7885021 33731973

[CIT0104] Vos J, Van Der Putten PEL, Birch CJ. Effect of nitrogen supply on leaf appearance, leaf growth, leaf nitrogen economy and photosynthetic capacity in maize (*Zea mays* L.). Field Crops Res 2005;93:64–73. https://doi.org/10.1016/j.fcr.2004.09.013

[CIT0105] Wang X, Wang L, Shangguan Z. Leaf gas exchange and fluorescence of two winter wheat varieties in response to drought stress and nitrogen supply. PLoS One 2016;11:e0165733. https://doi.org/10.1371/journal.pone.0165733 27802318 PMC5089754

[CIT0106] Wang Y, Sperry JS, Anderegg WRL et al A theoretical and empirical assessment of stomatal optimization modeling. New Phytol 2020;227:311–25. https://doi.org/10.1111/nph.16572 32248532

[CIT0107] Wang X, Müller C, Elliot J et al Global irrigation contribution to wheat and maize yield. Nat Commun 2021;12:1235. https://doi.org/10.1038/s41467-021-21498-5 33623028 PMC7902844

[CIT0108] Wang L, Ning S, Zheng W et al Performance analysis of two typical greenhouse lettuce production systems: commercial hydroponic production and traditional soil cultivation. Front Plant Sci 2023;14:1165856. https://doi.org/10.3389/fpls.2023.1165856 37469780 PMC10353484

[CIT0109] Wei Y et al Equipment and intelligent control system in aquaponics: a review. IEEE 2019;7:169306–26. Available at: https://doi.org/10.1109/ACCESS.2019.2953491

[CIT0110] Wong CE et al Seeing the lights for leafy greens in indoor vertical farming. Trends Food Sci Technol 2020;106:48–63. https://doi.org/10.1016/J.TIFS.2020.09.031

[CIT0111] Wood S. Generalized Additive Models: An Introduction With R, Second Edition (2nd ed.). New York, NY, USA: Chapman and Hall/CRC Press, 2017.

[CIT0112] Xu Z, Zhou G. Responses of leaf stomatal density to water status and its relationship with photosynthesis in a grass. J Exp Bot 2008;59:3317–25. https://doi.org/10.1093/jxb/ern185 18648104 PMC2529243

[CIT0113] Xu W, Cui K, Xu A et al Drought stress condition increases root to shoot ratio via alteration of carbohydrate partitioning and enzymatic activity in rice seedlings. Acta Physiol Plant 2015a;37:1–11. https://doi.org/10.1007/s11738-014-1760-0

[CIT0114] Xu Y, Ge J, Tian S et al Effects of water-saving irrigation practices and drought resistant rice variety on greenhouse gas emissions from a no-till paddy in the central lowlands of China. Sci Total Environ 2015b;505:1043–52. https://doi.org/10.1016/j.scitotenv.2014.10.073 25461105

[CIT0115] Yuan Z, Jiang S, Sheng H et al Human perturbation of the global phosphorus cycle: changes and consequences. Environ Sci Technol 2018;52:2438–50. https://doi.org/10.1021/acs.est.7b03910 29402084

[CIT0116] Zhang L et al Improvement of pea biomass and seed productivity by simultaneous increase of phloem and embryo loading with amino acids. Plant J 2015;81:134–46. https://doi.org/10.1111/TPJ.12716 25353986

[CIT0117] Zhang J et al Variation and evolution of C:N ratio among different organs enable plants to adapt to N-limited environments Global Dryland Ecosystem Programme (Global-DEP) view project ecosystem restoration following disturbances view project. Global Change Biol 2019;26:2534–43. https://doi.org/10.1111/gcb.14973 31873968

[CIT0118] Zhang X, Davidson EA, Mauzerall DL et al Managing nitrogen for sustainable development. Nature 2015;528:51–9. https://doi.org/10.1038/nature15743 26595273

[CIT0119] Zhang J, Guan K, Peng B et al Sustainable irrigation based on co-regulation of soil water supply and atmospheric evaporative demand. Nat Commun 2021;12:5549. https://doi.org/10.1038/s41467-021-25254-7 34545076 PMC8452748

[CIT0120] Zhao W, Liu L, Shen Q et al Effects of water stress on photosynthesis, yield, and water use efficiency in winter wheat. Water 2020;12:2127. https://doi.org/10.3390/w12082127

[CIT0121] Zhou R, Yu X, Ottosen C-O et al Drought stress had a predominant effect over heat stress on three tomato cultivars subjected to combined stress. BMC Plant Biol 2017;17:1–13. https://doi.org/10.1186/S12870-017-0974-x 28122507 PMC5264292

[CIT0122] Zörb C, Geilfus C-M, Dietz K-J. Salinity and crop yield. Plant Biol 21:2019: 31–38. https://doi.org/10.1111/plb.12884 30059606

