# Supplementary material for: Acclimation of functional traits leads to biomass increases in leafy green species grown in aquaponics
Source: AoB Plants. 2025 Jan 20;17(2):plaf005. doi: 10.1093/aobpla/plaf005 (PMC11851069; doi:10.1093/aobpla/plaf005)
Supplement: plaf005_suppl_Supplementary_Materials [file plaf005_suppl_supplementary_materials.pdf]

Supplemental Materials

Figures

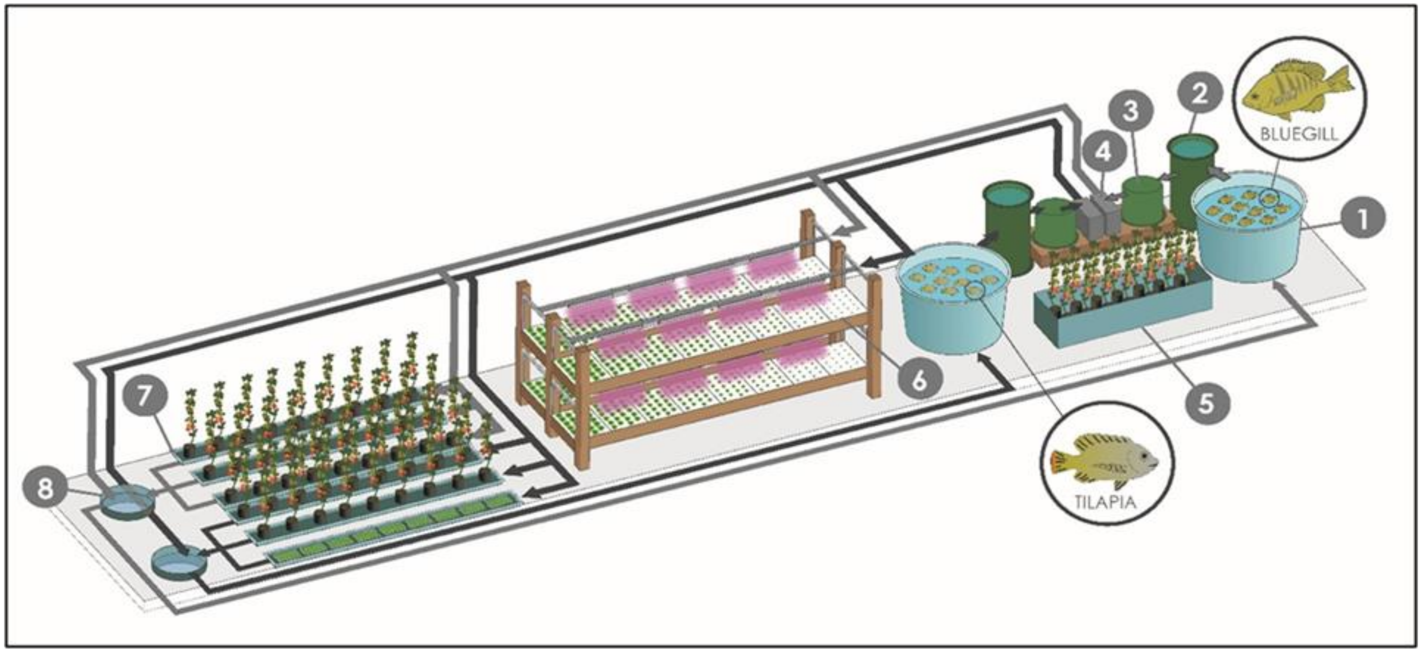

Figure S1. Shepherd University aquaponics laboratory. The laboratory contains two replicated experimental growing systems, one utilizing fish efflux from bluegill and the other tilapia. Each system consists of a (1) fish culture tank, (2) solid filtration tank, (3) biofilter, (4) distribution tank, (5) oxidizer grow-bed, (6) deep water culture beds with Styrofoam rafts for plant rearing, (7) vertical grow area, and a (8) sump where water is collected from different systems and returned to the fish culture tank (1). In this study, plants

grown in small pots with soil were floated on top of Styrofoam rafts, alongside plants growing in the deep water culture beds (6) using fish efflux from the tilapia tank.

Tables

Table S1. Comparison of biomass, stomatal anatomy, and stoichiometry for three leafy green species grown in aquaponics and soil treatments. Values of roots (g), shoots (g), the root to shoot ratio, stomatal density (# mm^{-2}), stomatal size (um^2), shoot $\delta^{13}C$ (‰), shoot and root nitrogen content ($N_{s,r}$ $g\ g^{-1}$), and shoot and root C:N represent the mean and ± 1 S.E.

Species	Treatment	Roots	Shoots	Root:Shoot	Stomatal Density	Stomatal Size	$\delta\ C^{13}$	N_s	N_r	Foliar C:N	Root C:N
Broccoli	Aquaponics	0.55 (0.069)	3.90 (0.415)	0.14 (0.010)	269.5 (2.84)	1.28 (0.012)	-38.2 (0.08)	0.056 (0.0007)	0.040 (0.0005)	6.3 (0.09)	10.0 (0.15)
	Soil	0.28 (0.010)	1.37 (0.041)	0.21 (0.008)	392.0 (7.20)	1.10 (0.012)	-37.8 (0.07)	0.012 (0.0006)	0.015 (0.0002)	30.5 (0.90)	25.5 (0.40)
Pak Choi	Aquaponics	1.09 (0.105)	7.73 (0.734)	0.15 (0.007)	451.0 (15.20)	1.87 (0.028)	-37.9 (0.24)	0.065 (0.0002)	0.038 (0.0014)	5.3 (0.30)	9.8 (0.21)
	Soil	0.54 (0.021)	3.58 (0.140)	0.15 (0.007)	436.4 (17.98)	1.90 (0.022)	-34.3 (0.18)	0.011 (0.0003)	0.019 (0.0004)	37.2 (1.09)	21.0 (0.42)
Salanova	Aquaponics	0.93 (0.038)	5.40 (0.177)	0.18 (0.012)	65.6 (1.94)	3.08 (0.030)	-33.6 (0.61)	0.058 (0.0002)	0.049 (0.0006)	5.8 (0.06)	8.0 (0.11)
	Soil	0.50 (0.029)	1.17 (0.060)	0.43 (0.017)	91.6 (3.77)	2.92 (0.035)	-33.0 (0.51)	0.015 (0.0011)	0.009 (0.0004)	25.7 (1.47)	46.4 (2.35)

Table S2. Comparison of leaf-level physiological parameters for three leafy green species grown in aquaponics and soil treatments. Values for net photosynthesis (A_n. μmols m^{-2} s^{-1}), transpiration (E, mmols m^{-2} s^{-1}), intrinsic water use efficiency (WUE_g, mmol CO_2 mol $H_2 0^{-1}$) from gas exchange, and stomatal conductance (g_s, mols m^{-2} s^{-1}) represent the mean ± 1 S.E.

Species	Treatment	A_n	E	WUE_g	g_s
Broccoli	Aquaponics	15.6 (0.353)	0.008 (0.0002)	16.9 (0.41)	0.942 (0.0242)
	Soil	11.2 (0.623)	0.004 (0.0002)	35.1 (2.14)	0.396 (0.0254)
Pak Choi	Aquaponics	18.8 (0.353)	0.006 (0.0001)	30.3 (1.50)	0.715 (0.0341)
	Soil	11.0 (0.205)	0.002 (0.0002)	65.7 (3.84)	0.224 (0.0194)
Salanova	Aquaponics	10.9 (0.354)	0.003 (0.0001)	30.8 (1.20)	0.387 (0.0202)
	Soil	4.3 (0.254)	0.001 (0.0001)	55.2 (2.39)	0.085 (0.0062)
